# Supplementary material for: Splice-Junction-Based Mapping of Alternative Isoforms in the Human Proteome
Source: Cell Rep. Author manuscript; Available in PMC 2020 Jan 15. (PMC6961840; doi:10.1016/j.celrep.2019.11.026)
Supplement: 2 [file NIHMS1546469-supplement-2.zip › DF1/PXD010154/Testis/Testis_3_CLTCL1_LQEHFQLQNLGINPANIGFSTLTMESDK.pdf]

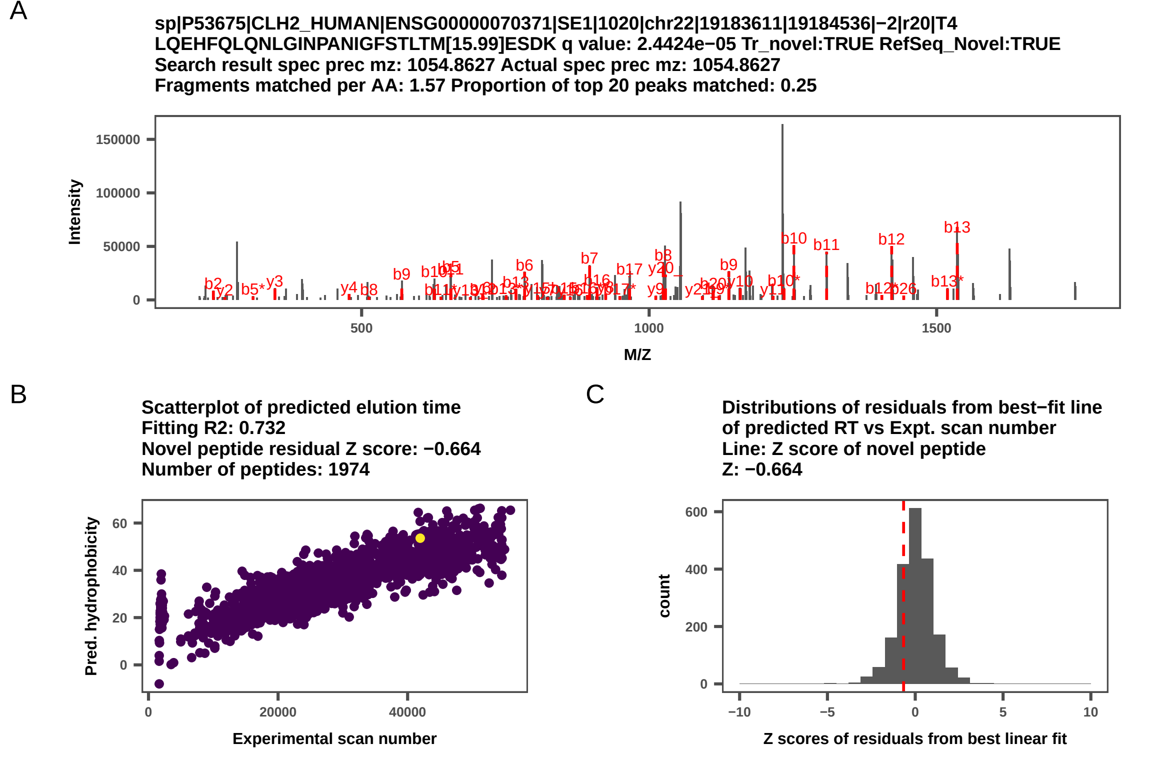
A
sp|P53675|CLH2_HUMAN|ENSG00000070371|SE1|1020|chr22|19183611|19184536|−2|r20|T4
LQEHFQLQNLGINPANIGFSTLTM[15.99]ESDK q value: 2.4424e−05 Tr_novel:TRUE RefSeq_Novel:TRUE
Search result spec prec mz: 1054.8627 Actual spec prec mz: 1054.8627
Fragments matched per AA: 1.57 Proportion of top 20 peaks matched: 0.25
Intensity
150000
100000
50000
0
500
1000
1500
M/Z
b2
y2
b5*
y3
y4
b8
b9
b6
b7
b17
b8
b9
b10
b11
b12
b13
b10*
b12*
b26
b13*
y9
y10
y11
B
Scatterplot of predicted elution time
Fitting R2: 0.732
Novel peptide residual Z score: −0.664
Number of peptides: 1974
Pred. hydrophobicity
60
40
20
0
20000
40000
Experimental scan number
C
Distributions of residuals from best−fit line
of predicted RT vs Expt. scan number
Line: Z score of novel peptide
Z: −0.664
count
600
400
200
0
−10
−5
0
5
10
Z scores of residuals from best linear fit
